# Genome Sequence of a Reassortant H5N1 Avian Influenza Virus Isolated from Domestic Green-Winged Teal

**DOI:** 10.1128/genomeA.00639-13

**Published:** 2013-08-15

**Authors:** Chaochao Xiong, Qian Liu, Quanjiao Chen, Jianjun Chen

**Affiliations:** Center for Emerging Infectious Diseases, Wuhan Institute of Virology, Chinese Academy of Sciences, Wuhan, Hubei, China

## Abstract

An avian influenza virus strain, A/domestic green-winged teal/Hunan/3450/2006(H5N1) (DGW-T3450), was isolated from domestic green-winged teals. Genome analysis demonstrated that DGW-T3450 is a novel reassortant strain. The hemagglutinin (HA) and neuraminidase (NA) genes of this strain originated from H5N1 viruses circulating in poultry, while its remaining genes are derived from multiple ancestors, including viruses like those that infect wild birds.

## GENOME ANNOUNCEMENT

H5N1 highly pathogenic avian influenza (HPAI) viruses have seriously affected the Asian poultry industry since their recurrence in 2003. In a previous study, we isolated two H5N1 viral strains, closely related to viruses circulating in chickens and ducks, from apparently healthy domestic green-winged teals (1). These results suggested that domestic green-winged teals, a type of wild duck bred for meat, may play an important role in the transmission of avian influenza virus.

In this study, an H5N1 virus, designated A/domestic green-winged teal/Hunan/3450/2006 (DGW-T3450), was isolated from domestic green-winged teals. We carried out reverse transcription (RT)-PCR using universal primers for influenza A virus and sequenced the entire viral genome (2). The full lengths of the gene segments are as follows: hemagglutinin (HA) gene, 1,776 nucleotides (nt); neuraminidase (NA) gene, 1,398 nt; nucleoprotein (NP) gene, 1,565 nt; polymerase PB1 and PB2 genes, 2,341 nt each; polymerase acidic protein (PA) gene, 2,233 nt; matrix (M) gene, 1,027 nt; and nonstructural (NS) protein gene, 890 nt.

Sequence analysis revealed that the nucleotide sequence of the HA gene of DGW-T3450 is homologous to that of the already identified strain A/environment/Hunan/1-8/2007(H5N1), sharing 99% nucleotide homology, while the NA gene sequence is most closely related to that of A/chicken/Sichuan/81/2005(H5N1). The NP gene nucleotide sequence shares approximately 98%  identity with that of H8N4 A/duck/Yangzhou/02/2005(H8N4). The PB1 and PB2 gene fragments are most closely related to H6N2 isolate A/duck/Guizhou/2773/2006(H6N2) and H6N2 isolate A/duck/Guizhou/1088/2007(H6N2), respectively, with both sharing 99% nucleotide identity. The PA and M genes share the greatest DNA sequence identities (over 99%) with H7N7 isolate A/mallard/Korea/GH170/2007(H7N7) and H3N6 isolate A/red crested pochard/Mongolia/1915/2006(H3N6), respectively. The NS gene nucleotide sequence is 99% similar to that of the H3N1 isolate A/whooper swan/Mongolia/1-21/2007(H3N1). These results indicate that the DGW-T3450 virus is a novel reassortant H5N1 virus, with its HA and NA genes derived from H5N1 viruses circulating in poultry and its remaining genes originating from multiple ancestors, including viruses like those that infect wild birds.

Based on the deduced amino acid sequence of the HA gene, DGW-T3450 possesses multiple basic amino acids at the connecting peptide between HA1 and HA2 (RRRGKR/G), a signal of high pathogenicity in chickens (3). The receptor-binding pocket retains the amino acid residues 222Gln and 224Gly in HA1, indicating an α2,3-linked sialic acid preference and a greater likelihood of avian infectivity (1, 4). No 627Lys or 701Asn mutations, which are associated with high virulence in mice (5, 6), are found in PB2. In addition, no 5-amino-acid (aa) deletion is present in the middle of the NS protein as in the dominant H5N1 found in southern China. The absence of an His274Tyr mutation in the DGW-T3450 NA protein indicates that DGW-T3450 may be sensitive to neuraminidase inhibitors (7).

In summary, DGW-T3450 is a novel reassortant avian influenza virus with its gene constellation derived from multiple ancestors, including viruses like those that infect wild birds. These results highlight the importance of surveillance at live poultry markets.

### Nucleotide sequence accession numbers.

The genome sequences of A/domestic green-winged teal/Hunan/3450/2006(H5N1) have been deposited in GenBank under accession numbers KC690153 to KC690160.
